# Di-μ-chlorido-bis­{[*N*,*N*′-dicyclo­hexyl-*N*′′,*N*′′-bis­(trimethyl­sil­yl)guanidinato-κ^2^ *N*,*N*′](tetra­hydro­furan-κ*O*)magnesium(II)}

**DOI:** 10.1107/S1600536811023865

**Published:** 2011-06-25

**Authors:** Jie Cheng

**Affiliations:** a School of Biological and Chemical Engineering, Ningbo Institute of Technology, Zhejiang University, Ningbo 315100, People’s Republic of China

## Abstract

The dinuclear title complex, [Mg_2_(C_19_H_40_N_3_Si_2_)_2_Cl_2_(C_4_H_8_O)_2_], lies on a center of inversion. The Mg^2+^ ions are bonded to a chelating *N*,*N*′-bonded guanidinate anion, a tetra­hydro­furan mol­ecule and two bridging chloride anions. The geometry of the resulting five-coordinated Mg^2+^ ion is a very distorted square-based pyramid with the O atom in the apical position.

## Related literature

For the synthesis of analogous metal-ligated complexes, see: Sánchez-Barba *et al.* (2006 ▶); Doring & Kempe (2009 ▶); Lyubov *et al.* (2007 ▶). For a review of the crystal structures of guanidinato-ligated metal complexes, see: Bailey & Pace (2001 ▶).
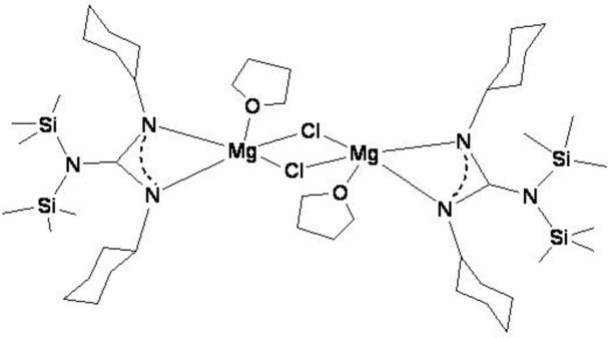


## Experimental

### 

#### Crystal data

[Mg_2_(C_19_H_40_N_3_Si_2_)_2_Cl_2_(C_4_H_8_O)_2_]
*M* *_r_* = 997.17
Triclinic, 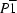

*a* = 8.7249 (3) Å
*b* = 11.0016 (5) Å
*c* = 16.8893 (8) Å
α = 79.487 (6)°
β = 75.211 (6)°
γ = 72.201 (5)°
*V* = 1482.80 (11) Å^3^
*Z* = 1
Mo *K*α radiation
μ = 0.25 mm^−1^
*T* = 223 K
0.60 × 0.30 × 0.22 mm

#### Data collection

Rigaku Saturn diffractometer
Absorption correction: multi-scan (*REQAB*; Jacobson, 1998 ▶) *T*_min_ = 0.340, *T*_max_ = 0.462
12201 measured reflections
5485 independent reflections
4175 reflections with *I* > 2σ(*I*)
*R*_int_ = 0.028

#### Refinement

*R*[*F* ^2^ > 2σ(*F* ^2^)] = 0.050
*wR*(*F* ^2^) = 0.141
*S* = 1.08
5485 reflections
287 parameters
2 restraints
H-atom parameters constrained
Δρ_max_ = 0.40 e Å^−3^
Δρ_min_ = −0.42 e Å^−3^

### 

Data collection: *CrystalClear* (Rigaku, 2000 ▶); cell refinement: *CrystalClear*; data reduction: *CrystalStructure* (Rigaku, 2000 ▶); program(s) used to solve structure: *SHELXS97* (Sheldrick, 2008 ▶); program(s) used to refine structure: *SHELXL97* (Sheldrick, 2008 ▶); molecular graphics: *SHELXTL* (Sheldrick, 2008 ▶); software used to prepare material for publication: *SHELXTL*.

## Supplementary Material

Crystal structure: contains datablock(s) I, global. DOI: 10.1107/S1600536811023865/hb5890sup1.cif

Structure factors: contains datablock(s) I. DOI: 10.1107/S1600536811023865/hb5890Isup2.hkl

Additional supplementary materials:  crystallographic information; 3D view; checkCIF report

## Figures and Tables

**Table 1:** Selected bond lengths (Å)

Mg1—O1	2.0334 (19)
Mg1—N2	2.0734 (18)
Mg1—N1	2.1247 (17)
Mg1—Cl1^i^	2.4946 (9)
Mg1—Cl1	2.4171 (9)

Symmetry code: (i) 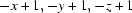.

**Fig. 1.**
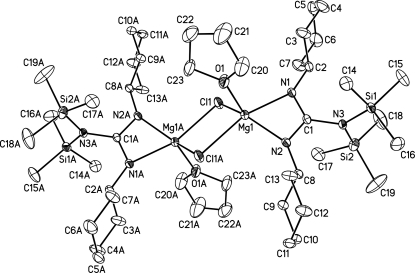
Molecular structure of the title compound with displacement elliposids drawn at the 30% probability level. Atoms with suffix A are generated by the symmetry operation (1–x, 1–y, 1–z).

## supplementary crystallographic information

### Comment

The title compound exists as a centrosymmetric dinuclear molecule in which each Mg^2+^ is five-coordinated by one bidentate guanidinato anion in *h*^2^-fashion, two chlorido anions, and one THF molecule to adopt a distorted pyramidal geometry (Fig. 1). The two {Mg(guanidinato)(THF)}^+^ moieties are connected by two chlorido anions in *m*^2^-mode.

### Experimental

The mono(guanidinato) yttrium dichloride complex was synthesized by the reaction of guanidinato lithium with one equivalent of YCl_3_ in THF according to a literature procedure. Treatment of the mono(guanidinato) yttrium dichloride complex with Mg(C_3_H_5_)Cl in 1:2 molar ratio in THF at room temperature, after recrystallization, afforded the title complex as colorless crystals. Colourless prisms were obtained from a saturated hexane solution at 243 K.

### Refinement

H-atoms were placed in calculated positions and were included in the refinement in the riding model approximation.

### Crystal data

**Table:**

[Mg_2_(C_19_H_40_N_3_Si_2_)_2_Cl_2_(C_4_H_8_O)_2_]	*Z* = 1
*M**_r_* = 997.17	*F*(000) = 544
Triclinic, *P*1	*D*_x_ = 1.117 Mg m^−^^3^
Hall symbol: -P 1	Mo *K*α radiation, λ = 0.71075 Å
*a* = 8.7249 (3) Å	Cell parameters from 6219 reflections
*b* = 11.0016 (5) Å	θ = 3.0–27.5°
*c* = 16.8893 (8) Å	µ = 0.25 mm^−^^1^
α = 79.487 (6)°	*T* = 223 K
β = 75.211 (6)°	Prism, colourless
γ = 72.201 (5)°	0.60 × 0.30 × 0.22 mm
*V* = 1482.80 (11) Å^3^	

### Data collection

**Table:**

Rigaku Saturn diffractometer	5485 independent reflections
Radiation source: fine-focus sealed tube	4175 reflections with *I* > 2σ(*I*)
graphite	*R*_int_ = 0.028
Detector resolution: 14.63 pixels mm^-1^	θ_max_ = 25.5°, θ_min_ = 3.0°
ω scans	*h* = −10→10
Absorption correction: multi-scan (REQAB; Jacobson, 1998)	*k* = −13→11
*T*_min_ = 0.340, *T*_max_ = 0.462	*l* = −20→18
12201 measured reflections	

### Refinement

**Table:**

Refinement on *F*^2^	Primary atom site location: structure-invariant direct methods
Least-squares matrix: full	Secondary atom site location: difference Fourier map
*R*[*F*^2^ > 2σ(*F*^2^)] = 0.050	Hydrogen site location: inferred from neighbouring sites
*wR*(*F*^2^) = 0.141	H-atom parameters constrained
*S* = 1.08	*w* = 1/[σ^2^(*F*_o_^2^) + (0.0855*P*)^2^] where *P* = (*F*_o_^2^ + 2*F*_c_^2^)/3
5485 reflections	(Δ/σ)_max_ = 0.001
287 parameters	Δρ_max_ = 0.40 e Å^−^^3^
2 restraints	Δρ_min_ = −0.42 e Å^−^^3^

### Special details

**Table:**

Geometry. All e.s.d.'s (except the e.s.d. in the dihedral angle between two l.s. planes) are estimated using the full covariance matrix. The cell e.s.d.'s are taken into account individually in the estimation of e.s.d.'s in distances, angles and torsion angles; correlations between e.s.d.'s in cell parameters are only used when they are defined by crystal symmetry. An approximate (isotropic) treatment of cell e.s.d.'s is used for estimating e.s.d.'s involving l.s. planes.
Refinement. Refinement of *F*^2^ against ALL reflections. The weighted *R*-factor *wR* and goodness of fit *S* are based on *F*^2^, conventional *R*-factors *R* are based on *F*, with *F* set to zero for negative *F*^2^. The threshold expression of *F*^2^ > σ(*F*^2^) is used only for calculating *R*-factors(gt) *etc*. and is not relevant to the choice of reflections for refinement. *R*-factors based on *F*^2^ are statistically about twice as large as those based on *F*, and *R*- factors based on ALL data will be even larger.

### Fractional atomic coordinates and isotropic or equivalent isotropic displacement parameters (Å^2^)

**Table:**

	*x*	*y*	*z*	*U*_iso_*/*U*_eq_	
Cl1	0.57869 (8)	0.59193 (6)	0.53865 (3)	0.0538 (2)	
Si1	0.19612 (8)	0.27490 (6)	0.89356 (3)	0.04007 (19)	
Si2	0.57738 (8)	0.19428 (7)	0.83044 (4)	0.0486 (2)	
Mg1	0.37458 (9)	0.47603 (7)	0.60061 (4)	0.0372 (2)	
O1	0.1567 (2)	0.6051 (2)	0.58704 (11)	0.0670 (6)	
N1	0.3601 (2)	0.47652 (18)	0.72806 (10)	0.0400 (4)	
N2	0.3613 (2)	0.30543 (17)	0.67452 (10)	0.0350 (4)	
N3	0.3771 (2)	0.27627 (17)	0.81889 (9)	0.0319 (4)	
C1	0.3639 (2)	0.3536 (2)	0.74099 (11)	0.0308 (4)	
C2	0.3718 (3)	0.5451 (2)	0.79118 (13)	0.0489 (6)	
H2	0.3730	0.4863	0.8431	0.059*	
C3	0.2249 (4)	0.6613 (3)	0.80647 (19)	0.0698 (8)	
H3A	0.1235	0.6336	0.8224	0.084*	
H3B	0.2207	0.7198	0.7553	0.084*	
C4	0.2330 (5)	0.7332 (4)	0.8742 (2)	0.0911 (12)	
H4A	0.1395	0.8102	0.8800	0.109*	
H4B	0.2250	0.6780	0.9268	0.109*	
C5	0.3926 (5)	0.7717 (3)	0.85416 (16)	0.0771 (10)	
H5A	0.3921	0.8377	0.8065	0.093*	
H5B	0.4000	0.8090	0.9011	0.093*	
C6	0.5393 (5)	0.6602 (4)	0.8355 (2)	0.0837 (10)	
H6A	0.5498	0.6008	0.8859	0.100*	
H6B	0.6385	0.6907	0.8174	0.100*	
C7	0.5280 (4)	0.5886 (3)	0.76892 (18)	0.0670 (8)	
H7A	0.5304	0.6449	0.7167	0.080*	
H7B	0.6238	0.5135	0.7611	0.080*	
C8	0.3507 (2)	0.1751 (2)	0.67767 (12)	0.0345 (5)	
H8	0.3583	0.1305	0.7336	0.041*	
C9	0.4891 (3)	0.0989 (2)	0.61505 (13)	0.0410 (5)	
H9A	0.4838	0.1429	0.5595	0.049*	
H9B	0.5956	0.0953	0.6258	0.049*	
C10	0.4761 (3)	−0.0373 (2)	0.61940 (14)	0.0461 (6)	
H10A	0.5638	−0.0821	0.5769	0.055*	
H10B	0.4926	−0.0838	0.6732	0.055*	
C11	0.3108 (3)	−0.0390 (2)	0.60709 (15)	0.0490 (6)	
H11A	0.3020	−0.0058	0.5500	0.059*	
H11B	0.3031	−0.1277	0.6167	0.059*	
C12	0.1706 (3)	0.0416 (3)	0.66506 (18)	0.0599 (7)	
H12A	0.1683	−0.0004	0.7216	0.072*	
H12B	0.0662	0.0469	0.6512	0.072*	
C13	0.1866 (3)	0.1767 (3)	0.66063 (18)	0.0534 (6)	
H13A	0.0963	0.2239	0.7011	0.064*	
H13B	0.1770	0.2220	0.6057	0.064*	
C14	0.0156 (3)	0.3832 (3)	0.85500 (17)	0.0582 (7)	
H14A	0.0309	0.4687	0.8390	0.087*	
H14B	−0.0829	0.3867	0.8981	0.087*	
H14C	0.0040	0.3510	0.8077	0.087*	
C15	0.1996 (4)	0.3282 (4)	0.99136 (16)	0.0809 (10)	
H15A	0.2800	0.2636	1.0181	0.121*	
H15B	0.0915	0.3401	1.0276	0.121*	
H15C	0.2293	0.4088	0.9794	0.121*	
C16	0.1665 (4)	0.1109 (3)	0.9186 (2)	0.0867 (11)	
H16A	0.1686	0.0788	0.8684	0.130*	
H16B	0.0612	0.1141	0.9563	0.130*	
H16C	0.2543	0.0543	0.9442	0.130*	
C17	0.7269 (3)	0.2293 (3)	0.73586 (18)	0.0684 (8)	
H17A	0.7127	0.1940	0.6905	0.103*	
H17B	0.8381	0.1907	0.7446	0.103*	
H17C	0.7085	0.3215	0.7229	0.103*	
C18	0.6256 (4)	0.2438 (5)	0.9186 (2)	0.1038 (14)	
H18A	0.6050	0.3364	0.9120	0.156*	
H18B	0.7405	0.2035	0.9203	0.156*	
H18C	0.5564	0.2172	0.9696	0.156*	
C19	0.6096 (5)	0.0173 (4)	0.8484 (4)	0.129 (2)	
H19A	0.5437	−0.0052	0.9016	0.193*	
H19B	0.7251	−0.0249	0.8477	0.193*	
H19C	0.5770	−0.0103	0.8054	0.193*	
C20	0.0019 (4)	0.6055 (4)	0.6432 (2)	0.0992 (13)	
H20A	0.0158	0.5861	0.7004	0.119*	
H20B	−0.0449	0.5418	0.6317	0.119*	
C21	−0.1037 (6)	0.7348 (6)	0.6292 (4)	0.172 (3)	
H21A	−0.2109	0.7309	0.6234	0.206*	
H21B	−0.1217	0.7818	0.6763	0.206*	
C22	−0.0259 (5)	0.8007 (4)	0.5551 (3)	0.1056 (13)	
H22A	−0.0022	0.8755	0.5680	0.127*	
H22B	−0.0981	0.8299	0.5155	0.127*	
C23	0.1279 (4)	0.7076 (4)	0.5207 (2)	0.0896 (11)	
H23A	0.1160	0.6748	0.4731	0.108*	
H23B	0.2192	0.7475	0.5035	0.108*	

### Atomic displacement parameters (Å^2^)

**Table:**

	*U*^11^	*U*^22^	*U*^33^	*U*^12^	*U*^13^	*U*^23^
Cl1	0.0806 (4)	0.0615 (4)	0.0290 (3)	−0.0396 (4)	−0.0038 (3)	−0.0054 (2)
Si1	0.0467 (4)	0.0415 (4)	0.0285 (3)	−0.0158 (3)	0.0035 (2)	−0.0051 (2)
Si2	0.0422 (4)	0.0536 (5)	0.0536 (4)	−0.0178 (3)	−0.0203 (3)	0.0075 (3)
Mg1	0.0451 (4)	0.0361 (4)	0.0264 (3)	−0.0095 (3)	−0.0043 (3)	−0.0013 (3)
O1	0.0567 (11)	0.0719 (14)	0.0464 (10)	0.0067 (10)	−0.0073 (8)	0.0108 (9)
N1	0.0589 (11)	0.0359 (11)	0.0257 (8)	−0.0166 (9)	−0.0045 (8)	−0.0043 (7)
N2	0.0435 (9)	0.0367 (11)	0.0259 (8)	−0.0128 (8)	−0.0048 (7)	−0.0068 (7)
N3	0.0367 (9)	0.0354 (10)	0.0248 (8)	−0.0143 (8)	−0.0046 (7)	−0.0015 (7)
C1	0.0288 (9)	0.0373 (13)	0.0249 (9)	−0.0097 (9)	−0.0020 (7)	−0.0041 (8)
C2	0.0848 (17)	0.0400 (14)	0.0270 (10)	−0.0288 (13)	−0.0077 (11)	−0.0023 (9)
C3	0.0805 (19)	0.060 (2)	0.0731 (18)	−0.0304 (16)	0.0093 (15)	−0.0344 (15)
C4	0.138 (3)	0.071 (2)	0.0680 (19)	−0.051 (2)	0.019 (2)	−0.0372 (17)
C5	0.149 (3)	0.059 (2)	0.0388 (13)	−0.057 (2)	−0.0124 (17)	−0.0067 (13)
C6	0.122 (3)	0.088 (3)	0.0692 (19)	−0.054 (2)	−0.0325 (19)	−0.0161 (18)
C7	0.0733 (18)	0.076 (2)	0.0661 (17)	−0.0309 (16)	−0.0113 (14)	−0.0298 (15)
C8	0.0399 (11)	0.0360 (13)	0.0280 (10)	−0.0122 (10)	−0.0033 (8)	−0.0067 (8)
C9	0.0394 (11)	0.0417 (14)	0.0380 (11)	−0.0068 (10)	−0.0025 (9)	−0.0104 (10)
C10	0.0527 (13)	0.0371 (14)	0.0386 (12)	−0.0003 (11)	−0.0034 (10)	−0.0091 (10)
C11	0.0583 (14)	0.0389 (14)	0.0511 (13)	−0.0129 (12)	−0.0058 (11)	−0.0177 (11)
C12	0.0471 (13)	0.0624 (19)	0.0775 (18)	−0.0236 (13)	0.0025 (13)	−0.0336 (14)
C13	0.0388 (12)	0.0507 (16)	0.0746 (17)	−0.0090 (11)	−0.0037 (12)	−0.0340 (13)
C14	0.0399 (12)	0.071 (2)	0.0605 (15)	−0.0193 (13)	0.0009 (11)	−0.0078 (13)
C15	0.0731 (19)	0.133 (3)	0.0381 (14)	−0.033 (2)	0.0053 (13)	−0.0294 (17)
C16	0.095 (2)	0.054 (2)	0.086 (2)	−0.0323 (17)	0.0359 (19)	−0.0026 (16)
C17	0.0346 (12)	0.091 (2)	0.0725 (18)	−0.0094 (14)	−0.0023 (12)	−0.0195 (16)
C18	0.0684 (19)	0.196 (5)	0.0614 (19)	−0.042 (2)	−0.0300 (16)	−0.017 (2)
C19	0.080 (2)	0.058 (2)	0.253 (6)	−0.0194 (19)	−0.087 (3)	0.045 (3)
C20	0.0543 (18)	0.118 (3)	0.092 (2)	−0.0068 (19)	−0.0064 (17)	0.028 (2)
C21	0.092 (3)	0.161 (5)	0.151 (5)	0.055 (3)	0.015 (3)	0.044 (4)
C22	0.095 (3)	0.083 (3)	0.117 (3)	0.011 (2)	−0.042 (2)	0.010 (2)
C23	0.090 (2)	0.087 (3)	0.067 (2)	−0.001 (2)	−0.0260 (17)	0.0263 (18)

### Geometric parameters (Å, °)

**Table:**

Si1—N3	1.7532 (16)	C9—H9A	0.9800
Si1—C14	1.849 (3)	C9—H9B	0.9800
Si1—C16	1.857 (3)	C10—C11	1.515 (3)
Si1—C15	1.861 (3)	C10—H10A	0.9800
Si2—N3	1.7472 (18)	C10—H10B	0.9800
Si2—C17	1.850 (3)	C11—C12	1.511 (3)
Si2—C18	1.857 (3)	C11—H11A	0.9800
Si2—C19	1.858 (4)	C11—H11B	0.9800
Mg1—O1	2.0334 (19)	C12—C13	1.523 (4)
Mg1—N2	2.0734 (18)	C12—H12A	0.9800
Mg1—N1	2.1247 (17)	C12—H12B	0.9800
Mg1—Cl1^i^	2.4946 (9)	C13—H13A	0.9800
Mg1—Cl1	2.4171 (9)	C13—H13B	0.9800
Mg1—Mg1^i^	3.5878 (13)	C14—H14A	0.9700
O1—C20	1.439 (4)	C14—H14B	0.9700
O1—C23	1.449 (3)	C14—H14C	0.9700
N1—C1	1.321 (3)	C15—H15A	0.9700
N1—C2	1.453 (3)	C15—H15B	0.9700
N2—C1	1.334 (3)	C15—H15C	0.9700
N2—C8	1.455 (3)	C16—H16A	0.9700
N3—C1	1.441 (2)	C16—H16B	0.9700
C2—C3	1.515 (4)	C16—H16C	0.9700
C2—C7	1.516 (4)	C17—H17A	0.9700
C2—H2	0.9900	C17—H17B	0.9700
C3—C4	1.531 (4)	C17—H17C	0.9700
C3—H3A	0.9800	C18—H18A	0.9700
C3—H3B	0.9800	C18—H18B	0.9700
C4—C5	1.517 (5)	C18—H18C	0.9700
C4—H4A	0.9800	C19—H19A	0.9700
C4—H4B	0.9800	C19—H19B	0.9700
C5—C6	1.489 (5)	C19—H19C	0.9700
C5—H5A	0.9800	C20—C21	1.455 (6)
C5—H5B	0.9800	C20—H20A	0.9800
C6—C7	1.524 (4)	C20—H20B	0.9800
C6—H6A	0.9800	C21—C22	1.445 (6)
C6—H6B	0.9800	C21—H21A	0.9800
C7—H7A	0.9800	C21—H21B	0.9800
C7—H7B	0.9800	C22—C23	1.471 (5)
C8—C13	1.525 (3)	C22—H22A	0.9800
C8—C9	1.527 (3)	C22—H22B	0.9800
C8—H8	0.9900	C23—H23A	0.9800
C9—C10	1.525 (3)	C23—H23B	0.9800
			
Mg1—Cl1—Mg1^i^	93.84 (3)	C13—C8—H8	108.6
N3—Si1—C14	110.04 (10)	C9—C8—H8	108.6
N3—Si1—C16	111.06 (12)	C10—C9—C8	111.38 (17)
C14—Si1—C16	108.07 (16)	C10—C9—H9A	109.4
N3—Si1—C15	111.90 (11)	C8—C9—H9A	109.4
C14—Si1—C15	108.22 (15)	C10—C9—H9B	109.4
C16—Si1—C15	107.41 (18)	C8—C9—H9B	109.4
N3—Si2—C17	109.83 (11)	H9A—C9—H9B	108.0
N3—Si2—C18	111.12 (14)	C11—C10—C9	112.2 (2)
C17—Si2—C18	108.39 (16)	C11—C10—H10A	109.2
N3—Si2—C19	112.06 (12)	C9—C10—H10A	109.2
C17—Si2—C19	107.5 (2)	C11—C10—H10B	109.2
C18—Si2—C19	107.8 (2)	C9—C10—H10B	109.2
O1—Mg1—N2	116.59 (8)	H10A—C10—H10B	107.9
O1—Mg1—N1	100.44 (8)	C12—C11—C10	111.19 (19)
N2—Mg1—N1	64.10 (7)	C12—C11—H11A	109.4
O1—Mg1—Cl1	104.41 (7)	C10—C11—H11A	109.4
N2—Mg1—Cl1	138.03 (6)	C12—C11—H11B	109.4
N1—Mg1—Cl1	101.29 (6)	C10—C11—H11B	109.4
O1—Mg1—Cl1^i^	91.66 (6)	H11A—C11—H11B	108.0
N2—Mg1—Cl1^i^	100.61 (5)	C11—C12—C13	112.2 (2)
N1—Mg1—Cl1^i^	163.61 (6)	C11—C12—H12A	109.2
Cl1—Mg1—Cl1^i^	86.16 (3)	C13—C12—H12A	109.2
O1—Mg1—C1	113.31 (7)	C11—C12—H12B	109.2
N2—Mg1—C1	32.25 (7)	C13—C12—H12B	109.2
N1—Mg1—C1	31.92 (7)	H12A—C12—H12B	107.9
Cl1—Mg1—C1	122.25 (5)	C12—C13—C8	111.8 (2)
Cl1^i^—Mg1—C1	132.33 (6)	C12—C13—H13A	109.3
O1—Mg1—Mg1^i^	100.82 (6)	C8—C13—H13A	109.3
N2—Mg1—Mg1^i^	128.97 (6)	C12—C13—H13B	109.3
N1—Mg1—Mg1^i^	143.03 (6)	C8—C13—H13B	109.3
Cl1—Mg1—Mg1^i^	43.93 (2)	H13A—C13—H13B	107.9
Cl1^i^—Mg1—Mg1^i^	42.236 (19)	Si1—C14—H14A	109.5
C1—Mg1—Mg1^i^	145.86 (6)	Si1—C14—H14B	109.5
C20—O1—C23	108.6 (2)	H14A—C14—H14B	109.5
C20—O1—Mg1	123.90 (18)	Si1—C14—H14C	109.5
C23—O1—Mg1	127.48 (18)	H14A—C14—H14C	109.5
C1—N1—C2	122.68 (17)	H14B—C14—H14C	109.5
C1—N1—Mg1	89.82 (12)	Si1—C15—H15A	109.5
C2—N1—Mg1	146.53 (14)	Si1—C15—H15B	109.5
C1—N2—C8	122.48 (17)	H15A—C15—H15B	109.5
C1—N2—Mg1	91.70 (13)	Si1—C15—H15C	109.5
C8—N2—Mg1	145.82 (13)	H15A—C15—H15C	109.5
C1—N3—Si2	115.52 (12)	H15B—C15—H15C	109.5
C1—N3—Si1	118.56 (13)	Si1—C16—H16A	109.5
Si2—N3—Si1	125.91 (10)	Si1—C16—H16B	109.5
N1—C1—N2	114.12 (17)	H16A—C16—H16B	109.5
N1—C1—N3	123.27 (17)	Si1—C16—H16C	109.5
N2—C1—N3	122.56 (18)	H16A—C16—H16C	109.5
N1—C1—Mg1	58.25 (10)	H16B—C16—H16C	109.5
N2—C1—Mg1	56.05 (10)	Si2—C17—H17A	109.5
N3—C1—Mg1	173.59 (14)	Si2—C17—H17B	109.5
N1—C2—C3	111.2 (2)	H17A—C17—H17B	109.5
N1—C2—C7	112.17 (19)	Si2—C17—H17C	109.5
C3—C2—C7	108.8 (2)	H17A—C17—H17C	109.5
N1—C2—H2	108.2	H17B—C17—H17C	109.5
C3—C2—H2	108.2	Si2—C18—H18A	109.5
C7—C2—H2	108.2	Si2—C18—H18B	109.5
C2—C3—C4	112.0 (3)	H18A—C18—H18B	109.5
C2—C3—H3A	109.2	Si2—C18—H18C	109.5
C4—C3—H3A	109.2	H18A—C18—H18C	109.5
C2—C3—H3B	109.2	H18B—C18—H18C	109.5
C4—C3—H3B	109.2	Si2—C19—H19A	109.5
H3A—C3—H3B	107.9	Si2—C19—H19B	109.5
C5—C4—C3	111.0 (2)	H19A—C19—H19B	109.5
C5—C4—H4A	109.4	Si2—C19—H19C	109.5
C3—C4—H4A	109.4	H19A—C19—H19C	109.5
C5—C4—H4B	109.4	H19B—C19—H19C	109.5
C3—C4—H4B	109.4	O1—C20—C21	105.0 (3)
H4A—C4—H4B	108.0	O1—C20—H20A	110.7
C6—C5—C4	111.9 (3)	C21—C20—H20A	110.7
C6—C5—H5A	109.2	O1—C20—H20B	110.7
C4—C5—H5A	109.2	C21—C20—H20B	110.7
C6—C5—H5B	109.2	H20A—C20—H20B	108.8
C4—C5—H5B	109.2	C22—C21—C20	109.2 (3)
H5A—C5—H5B	107.9	C22—C21—H21A	109.8
C5—C6—C7	112.3 (3)	C20—C21—H21A	109.8
C5—C6—H6A	109.1	C22—C21—H21B	109.8
C7—C6—H6A	109.1	C20—C21—H21B	109.8
C5—C6—H6B	109.1	H21A—C21—H21B	108.3
C7—C6—H6B	109.1	C21—C22—C23	106.6 (3)
H6A—C6—H6B	107.9	C21—C22—H22A	110.4
C2—C7—C6	111.8 (2)	C23—C22—H22A	110.4
C2—C7—H7A	109.3	C21—C22—H22B	110.4
C6—C7—H7A	109.3	C23—C22—H22B	110.4
C2—C7—H7B	109.3	H22A—C22—H22B	108.6
C6—C7—H7B	109.3	O1—C23—C22	105.1 (3)
H7A—C7—H7B	107.9	O1—C23—H23A	110.7
N2—C8—C13	110.59 (19)	C22—C23—H23A	110.7
N2—C8—C9	112.16 (16)	O1—C23—H23B	110.7
C13—C8—C9	108.22 (17)	C22—C23—H23B	110.7
N2—C8—H8	108.6	H23A—C23—H23B	108.8
			
Mg1^i^—Cl1—Mg1—O1	−90.73 (7)	Mg1—N2—C1—N1	4.89 (18)
Mg1^i^—Cl1—Mg1—N2	101.63 (9)	C8—N2—C1—N3	7.9 (3)
Mg1^i^—Cl1—Mg1—N1	165.26 (7)	Mg1—N2—C1—N3	−172.51 (15)
Mg1^i^—Cl1—Mg1—Cl1^i^	0.0	C8—N2—C1—Mg1	−179.6 (2)
Mg1^i^—Cl1—Mg1—C1	139.09 (7)	Si2—N3—C1—N1	−91.9 (2)
N2—Mg1—O1—C20	20.7 (3)	Si1—N3—C1—N1	86.6 (2)
N1—Mg1—O1—C20	−45.5 (3)	Si2—N3—C1—N2	85.2 (2)
Cl1—Mg1—O1—C20	−150.1 (3)	Si1—N3—C1—N2	−96.2 (2)
Cl1^i^—Mg1—O1—C20	123.4 (3)	Si2—N3—C1—Mg1	9.8 (13)
C1—Mg1—O1—C20	−14.8 (3)	Si1—N3—C1—Mg1	−171.7 (12)
Mg1^i^—Mg1—O1—C20	165.0 (3)	O1—Mg1—C1—N1	−71.43 (15)
N2—Mg1—O1—C23	−157.5 (3)	N2—Mg1—C1—N1	−174.75 (19)
N1—Mg1—O1—C23	136.3 (3)	Cl1—Mg1—C1—N1	54.89 (14)
Cl1—Mg1—O1—C23	31.7 (3)	Cl1^i^—Mg1—C1—N1	172.78 (11)
Cl1^i^—Mg1—O1—C23	−54.8 (3)	Mg1^i^—Mg1—C1—N1	108.96 (15)
C1—Mg1—O1—C23	167.0 (3)	O1—Mg1—C1—N2	103.33 (14)
Mg1^i^—Mg1—O1—C23	−13.3 (3)	N1—Mg1—C1—N2	174.75 (19)
O1—Mg1—N1—C1	117.72 (13)	Cl1—Mg1—C1—N2	−130.35 (11)
N2—Mg1—N1—C1	3.11 (11)	Cl1^i^—Mg1—C1—N2	−12.46 (14)
Cl1—Mg1—N1—C1	−135.13 (12)	Mg1^i^—Mg1—C1—N2	−76.29 (15)
Cl1^i^—Mg1—N1—C1	−19.2 (3)	O1—Mg1—C1—N3	−177.1 (12)
Mg1^i^—Mg1—N1—C1	−118.06 (13)	N2—Mg1—C1—N3	79.5 (13)
O1—Mg1—N1—C2	−75.3 (3)	N1—Mg1—C1—N3	−105.7 (13)
N2—Mg1—N1—C2	170.1 (3)	Cl1—Mg1—C1—N3	−50.8 (13)
Cl1—Mg1—N1—C2	31.8 (3)	Cl1^i^—Mg1—C1—N3	67.1 (13)
Cl1^i^—Mg1—N1—C2	147.7 (3)	Mg1^i^—Mg1—C1—N3	3.3 (13)
C1—Mg1—N1—C2	167.0 (4)	C1—N1—C2—C3	−123.4 (2)
Mg1^i^—Mg1—N1—C2	48.9 (3)	Mg1—N1—C2—C3	72.2 (3)
O1—Mg1—N2—C1	−92.06 (13)	C1—N1—C2—C7	114.6 (2)
N1—Mg1—N2—C1	−3.08 (11)	Mg1—N1—C2—C7	−49.9 (4)
Cl1—Mg1—N2—C1	74.54 (14)	N1—C2—C3—C4	178.7 (2)
Cl1^i^—Mg1—N2—C1	170.66 (11)	C7—C2—C3—C4	−57.3 (3)
Mg1^i^—Mg1—N2—C1	135.47 (11)	C2—C3—C4—C5	55.8 (4)
O1—Mg1—N2—C8	87.3 (3)	C3—C4—C5—C6	−52.8 (4)
N1—Mg1—N2—C8	176.3 (3)	C4—C5—C6—C7	53.0 (4)
Cl1—Mg1—N2—C8	−106.1 (2)	N1—C2—C7—C6	−179.9 (3)
Cl1^i^—Mg1—N2—C8	−10.0 (3)	C3—C2—C7—C6	56.7 (3)
C1—Mg1—N2—C8	179.4 (3)	C5—C6—C7—C2	−55.7 (4)
Mg1^i^—Mg1—N2—C8	−45.1 (3)	C1—N2—C8—C13	113.1 (2)
C17—Si2—N3—C1	−0.02 (19)	Mg1—N2—C8—C13	−66.2 (3)
C18—Si2—N3—C1	119.9 (2)	C1—N2—C8—C9	−126.0 (2)
C19—Si2—N3—C1	−119.5 (2)	Mg1—N2—C8—C9	54.7 (3)
C17—Si2—N3—Si1	−178.41 (14)	N2—C8—C9—C10	179.88 (18)
C18—Si2—N3—Si1	−58.5 (2)	C13—C8—C9—C10	−57.9 (2)
C19—Si2—N3—Si1	62.1 (2)	C8—C9—C10—C11	56.5 (2)
C14—Si1—N3—C1	−1.54 (18)	C9—C10—C11—C12	−52.5 (3)
C16—Si1—N3—C1	118.1 (2)	C10—C11—C12—C13	52.2 (3)
C15—Si1—N3—C1	−121.89 (19)	C11—C12—C13—C8	−56.2 (3)
C14—Si1—N3—Si2	176.82 (13)	N2—C8—C13—C12	−178.95 (19)
C16—Si1—N3—Si2	−63.56 (19)	C9—C8—C13—C12	57.8 (3)
C15—Si1—N3—Si2	56.46 (19)	C23—O1—C20—C21	−22.1 (5)
C2—N1—C1—N2	−176.27 (19)	Mg1—O1—C20—C21	159.4 (4)
Mg1—N1—C1—N2	−4.77 (17)	O1—C20—C21—C22	11.9 (7)
C2—N1—C1—N3	1.1 (3)	C20—C21—C22—C23	2.5 (7)
Mg1—N1—C1—N3	172.61 (16)	C20—O1—C23—C22	23.7 (4)
C2—N1—C1—Mg1	−171.5 (2)	Mg1—O1—C23—C22	−157.8 (3)
C8—N2—C1—N1	−174.70 (18)	C21—C22—C23—O1	−15.7 (6)

Symmetry codes: (i) −*x*+1, −*y*+1, −*z*+1.
